# Genome-Wide Identification and Analysis of bZIP Transcription Factors in *Coptis chinensis* Reveals Their Regulatory Roles in Stress Responses

**DOI:** 10.3390/ijms27010431

**Published:** 2025-12-31

**Authors:** Wuke Wei, Zijian Le, Lianan Guo, Rangyu Mo, Yu Wang, Yuan Pan

**Affiliations:** 1 Chongqing Academy of Chinese Materia Medica, Chongqing 400065, China; sahsw2@outlook.com (W.W.); guolianan@126.com (L.G.);; 2 Ministry of Education Key Laboratory of Chinese Medicinal Resource from Lingnan, School of Pharmaceutical Sciences, Guangzhou University of Chinese Medicine, Guangzhou 510006, China; 3 School of Life Sciences, Southwest University, Chongqing 400715, China; lezijian0917@outlook.com; 4 School of Future Technology, University of Chinese Academy of Sciences, Beijing 100049, China

**Keywords:** *Coptis chinensis*, bZIP transcription factors, genome-wide analysis, abiotic stress, gene expression

## Abstract

The basic leucine zipper (bZIP) transcription factors play crucial roles in plant growth and stress adaptation. However, a comprehensive genome-wide analysis of this family has been lacking in the medicinal plant *Coptis chinensis*. In this study, we identified 55 bZIP genes (*CcbZIPs*) from the *C. chinensis* genome and systematically classified them into 12 subfamilies through phylogenetic analysis with *Arabidopsis thaliana*. Notably, subfamilies L and O were absent, while two orphan genes were discovered, indicating lineage-specific evolution. Expression profiling revealed that numerous *CcbZIPs* respond dynamically to temperature and light stresses in a tissue-specific manner. These findings provide a foundation for understanding the regulatory roles of CcbZIPs in environmental adaptation and secondary metabolism, offering potential genetic targets for future breeding aimed at improving stress tolerance and medicinal compound production in *C. chinensis*.

## 1. Introduction

Transcription factors (TFs) are central regulators of gene expression, governing diverse processes in plant growth, development, and adaptation to environmental stresses [1,2]. Among various TF families, the basic leucine zipper (bZIP) family represents one of the largest and most evolutionarily conserved groups in eukaryotes [3]. Typical bZIP proteins are characterized by two functional domains: an N-terminal basic region that facilitates sequence-specific DNA binding, and a C-terminal leucine zipper domain responsible for dimerization [4]. This structure enables bZIP TFs to recognize a variety of DNA motifs—such as G-box (CACGTG), A-box (TACGTA), and C-box (GACGTC)—thereby regulating distinct transcriptional programs involved in numerous signaling pathways [1,5]. Functionally, bZIPs participate in a wide spectrum of physiological events, including embryogenesis, photomorphogenesis, flowering time control, and responses to biotic and abiotic stresses, underscoring their broad regulatory significance [6,7].

Genome-wide studies have systematically identified bZIP members across numerous plant species, revealing both conserved and lineage-specific evolutionary patterns [8]. In the model plant *Arabidopsis thaliana*, 78 bZIP genes have been classified into 13 subfamilies, with many members implicated in abscisic acid (ABA) signaling, pathogen defense, and energy homeostasis [1]. Similarly, in horticultural and medicinal plants such as litchi [9], wheat [10], rice [11], maize [12], tomato [13], potato [14], banana [15], cucumber [16], sesame [17], pomegranate [18], etc. bZIP TFs have been linked to fruit senescence, stress tolerance, and the biosynthesis of specialized metabolites like terpenoids [19]. Notably, bZIPs are increasingly recognized as key regulators of secondary metabolism in medicinal plants. For instance, AabZIP1, AabZIP9, and AaTGA6 in Artemisia annua positively regulate the biosynthesis of the antimalarial compound artemisinin [20,21,22], while bZIPs in *Carthamus tinctorius* modulate flavonoid biosynthesis [23]. These findings highlight the potential of engineering bZIP TFs to enhance the production of valuable phytochemicals [24].

As integrators of environmental signals, bZIP TFs also play pivotal roles in light and temperature response pathways. They participate in photomorphogenesis through HY5-mediated signaling and in temperature stress responses via both ABA-dependent and independent mechanisms [25]. Examples include MdHY5, which regulates light-induced anthocyanin biosynthesis in apple, and OsbZIP52, which enhances cold tolerance in rice [19,26]. These studies position bZIP TFs as critical hubs within stress-responsive networks, linking environmental perception to metabolic reprogramming [27].

*Coptis chinensis* (Chinese goldthread), a perennial herb of the Ranunculaceae family, is a renowned medicinal plant whose rhizomes accumulate bioactive isoquinoline alkaloids—such as berberine, palmatine, and coptisine—which exhibit antimicrobial, anti-inflammatory, and anticancer properties [28]. Environmental factors, particularly light and temperature, are known to influence both growth and alkaloid accumulation in medicinal plants [29,30]. However, despite its medicinal significance, the molecular mechanisms governing the biosynthesis and regulation of these valuable compounds remain poorly characterized. Moreover, how abiotic signals are perceived and transduced into transcriptional reprogramming—specifically through bZIP TFs—has not been systematically investigated in *C. chinensis*.

Therefore, to bridge this knowledge gap, we performed a genome-wide identification and analysis of the bZIP family in *C. chinensis*. This study aims to characterize the phylogenetic relationships, gene structures, conserved motifs, and chromosomal distributions of CcbZIP genes. Furthermore, we analyzed their expression patterns under different temperature and light conditions. Our findings provide a foundational resource for understanding the regulatory roles of CcbZIP TFs in the environmental adaptation and secondary metabolism of this medically important species.

## 2. Results

### 2.1. Genome-Wide Identification, Classification and Phylogenetic Analysis of CcbZIP Genes

Through a combination of BLASTP and HMMER searches against the *Coptis chinensis* genome, we identified 55 non-redundant proteins containing the characteristic bZIP domain. These genes were systematically renamed from CcbZIP1 to CcbZIP55 based on their ascending physical locations on the chromosomes (Table 1). The encoded proteins exhibited considerable diversity in their physicochemical properties. Their lengths ranged from 81 to 893 amino acids, corresponding to molecular weights from approximately 9.56 to 93.52 kDa. The theoretical isoelectric points (pI) varied from 4.89 to 11.44, with 21 proteins (38.2%) being acidic (pI < 7) and the remainder basic. All proteins were predicted to be hydrophilic, with grand average of hydropathy (GRAVY) values between −0.44 and 0.023. Subcellular localization predictions strongly indicated that the vast majority are nuclear proteins, consistent with their putative function as transcription factors [31].

**Figure 1:**
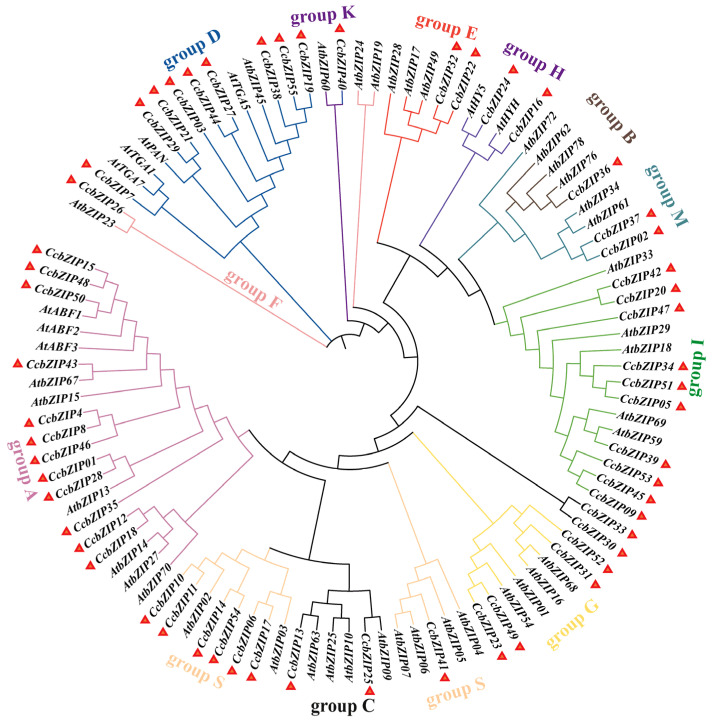
Phylogenetic tree of the bZIP gene family in *Coptis chinensis* and *A. thaliana*. The maximum likelihood (ML) phylogenetic tree was constructed using MEGA7.0. Subfamilies are indicated by different colored arcs. Bootstrap values from 1000 replicates are shown at key nodes. The triangle represents the *CcbZIP* genes.

To elucidate the evolutionary relationships within the bZIP family, a phylogenetic tree was constructed using the full-length amino acid sequences of the 55 CcbZIPs and 50 well-characterized AtbZIPs from *Arabidopsis thaliana* (Figure 1). The CcbZIP proteins were classified into subfamilies based on their clustering with established *A. thaliana* orthologs. This analysis classified the 55 members into 12 subfamilies: A, B, C, D, E, F, G, H, I, K, M, and S. Notably, subfamilies L and O, which are present in *A. thaliana*, were absent in *C. chinensis*. Subfamilies A and I were the most expanded, containing 12 and 10 members, respectively, followed by D (9), S (7), G (4), C (2), M (2), H (2), E (2), while B, F, and K each contained a single member. Two genes, *CcbZIP30* and *CcbZIP33*, did not cluster with any defined subfamily and were designated as orphan genes, suggesting potential lineage-specific evolution [32].

### 2.2. Gene Structure and Conserved Domain Analysis

**Figure 2:**
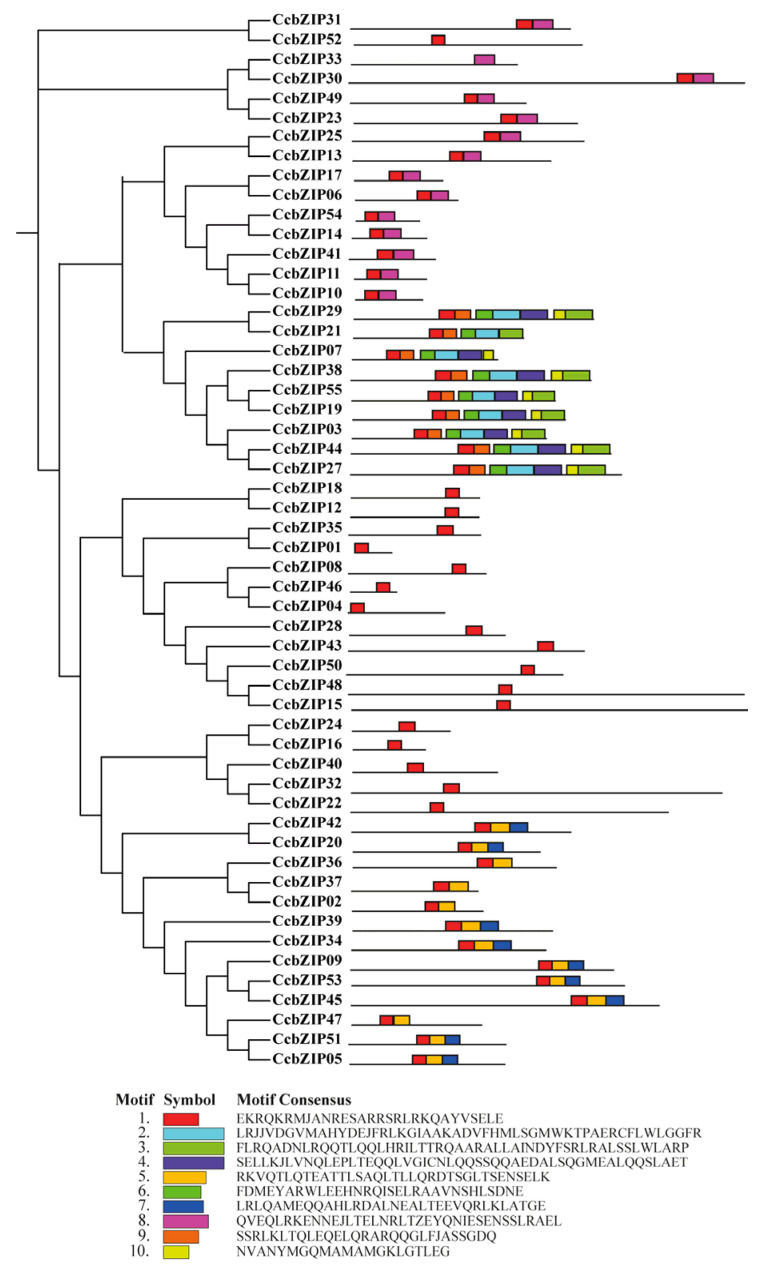
Distribution and architecture of conserved motifs in CcbZIP proteins. The motif analysis was performed using the MEME suite, identifying 10 distinct motifs (numbered 1–10), each represented by a unique color.

**Figure 3:**
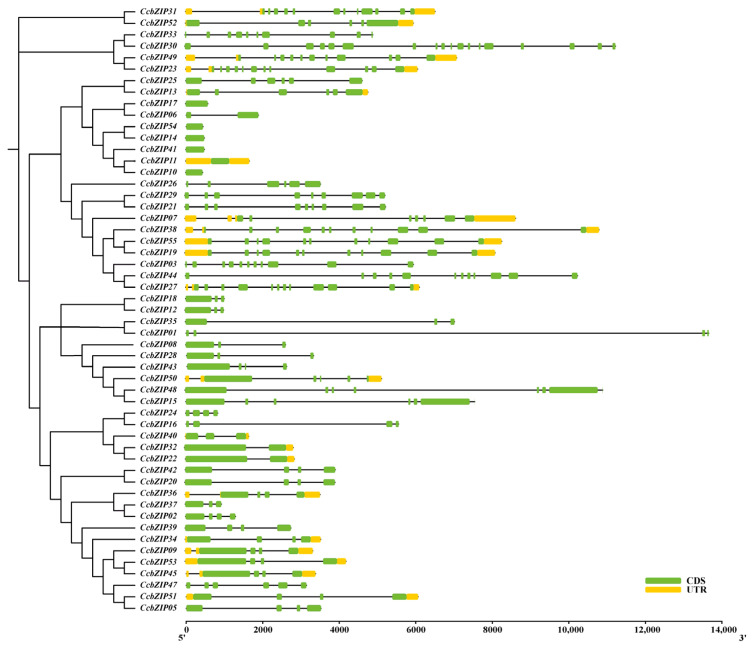
Exon–intron structure of CcbZIP genes. Gene structures are shown aligned with the phylogenetic groups. Exons and untranslated regions (UTRs) are represented by green and yellow boxes, respectively; black lines indicate introns.

To gain deeper insights into the structural characteristics and potential functional diversification of the CcbZIP family, we systematically analyzed their conserved motifs, gene structures, and domain architectures. As shown in Figure 2, conserved motif analysis identified 10 distinct motifs (Motif 1–10) distributed across the CcbZIP proteins. The composition and sequential order of these motifs were highly conserved within members of the same phylogenetic subfamily but exhibited clear variation between different subfamilies. For instance, Subfamily A members commonly shared Motifs 1, 2, 3, and 4, whereas Subfamily I was characterized by a distinct set including Motifs 1, 5, and 6. This specific motif architecture strongly supports the reliability of our phylogenetic classification and suggests potential functional specialization among subfamilies. Analysis of the exon–intron structures further revealed substantial structural diversity (Figure 3). Genes clustered within the same subfamily generally possessed similar exon–intron organizations, reinforcing their evolutionary relationships. Notably, members of the expanded subfamilies A and I typically contained multiple introns, indicating structural complexity. In contrast, several genes from other subfamilies exhibited fewer introns or intron-less structures. This structural variation provides genomic evidence for the divergent evolution of the CcbZIP family.

**Figure 4:**
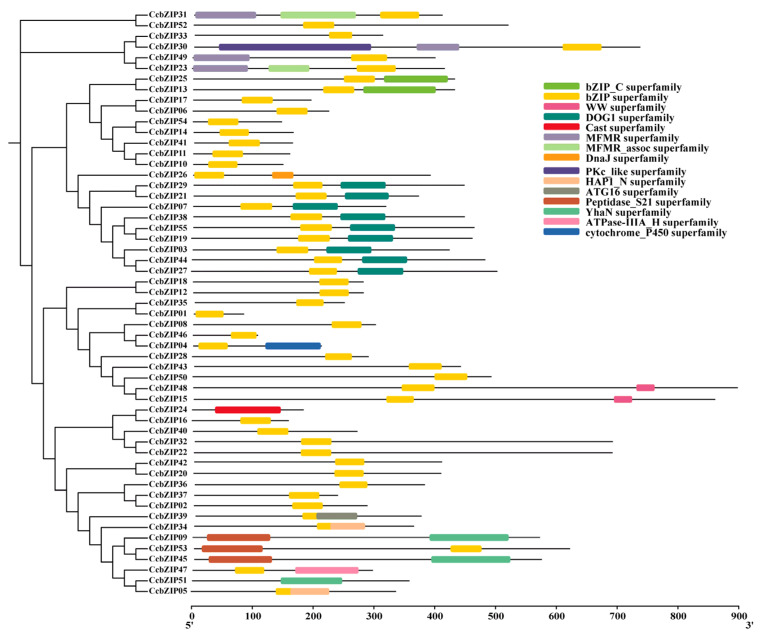
Conserved domain architecture of CcbZIP proteins. Protein domains were identified using the NCBI Conserved Domain Database. The bZIP domain, characteristic of this transcription factor family, is consistently present across all members and highlighted in distinct colors along with other identified functional domains.

Furthermore, examination of the conserved domain architecture confirmed the presence of the characteristic bZIP domain in all 55 CcbZIP proteins (Figure 4). Beyond this core domain, numerous members harbored additional functional domains, such as regulatory or protein–protein interaction domains, which were often specific to certain subfamilies. The diversity in domain composition underscores the functional versatility within this transcription factor family and supports the notion of subfamily-specific regulatory roles acquired during evolution. Collectively, the integrated analysis of motifs, gene structures, and domains reveals a strong correlation between phylogenetic grouping and structural features, providing a comprehensive genomic foundation for understanding the functional evolution of the bZIP family in *C. chinensis*.

### 2.3. Chromosomal Distribution, Gene Duplications and Synteny Analysis of CcbZIPs

**Figure 5:**
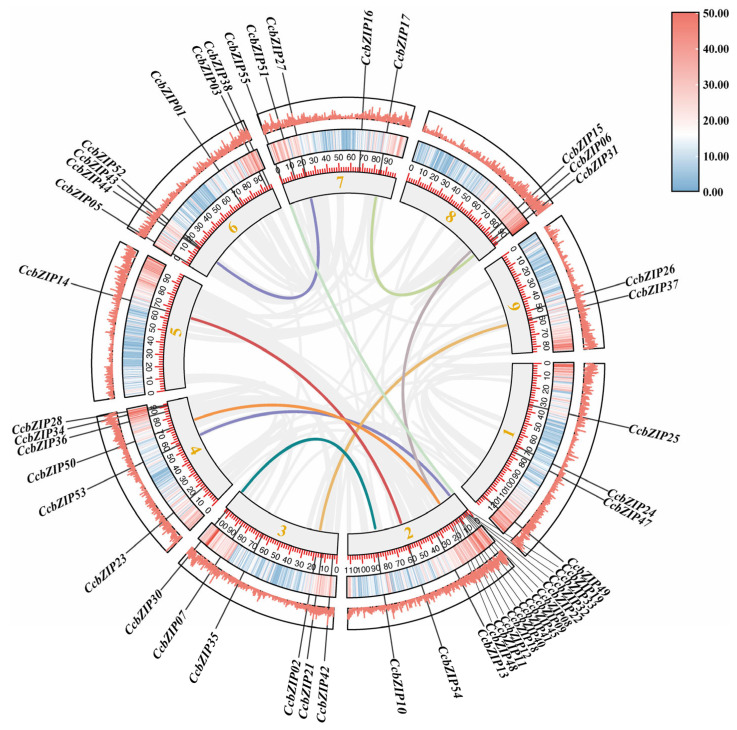
Chromosome positions and synteny analysis of *CcbZIP* genes. The physical positions of CcbZIP genes on the nine chromosomes of *C. chinensis* are shown. Genes are labeled on the right side of each chromosome. Interspecific syntenic blocks are color-coded to indicate homologous relationships between genes from different species, for visual clarity.

The genomic locations of the 55 CcbZIP genes were mapped onto the nine chromosomes of *C. chinensis* (Figure 5). The distribution was uneven, with chromosomes 1, 2, 3, 4, 6, 7, and 8 harboring multiple genes, while chromosome 5 contained only a single gene (CcbZIP14). To understand the evolutionary mechanisms underlying the expansion of the CcbZIP family, we performed synteny analysis. Several conserved syntenic blocks were identified within the *C. chinensis* genome, indicative of segmental duplication events. Significant collinearity was observed between specific chromosome pairs, including Chr1-Chr4, Chr2-Chr4, Chr2-Chr7, and Chr6-Chr7 (highlighted by lines in Figure 5), suggesting these events have been crucial in the evolution of this gene family. Tandem duplication events were also detected, with relevant genes highlighted in Figure 5. These findings suggest that both segmental and tandem duplications have contributed to the expansion of the bZIP family in *C. chinensis*, a pattern consistent with the evolution of gene families in other plant species [33].

### 2.4. Expression Patterns of CcbZIP Genes Under Different Temperature and Light Conditions

**Figure 6:**
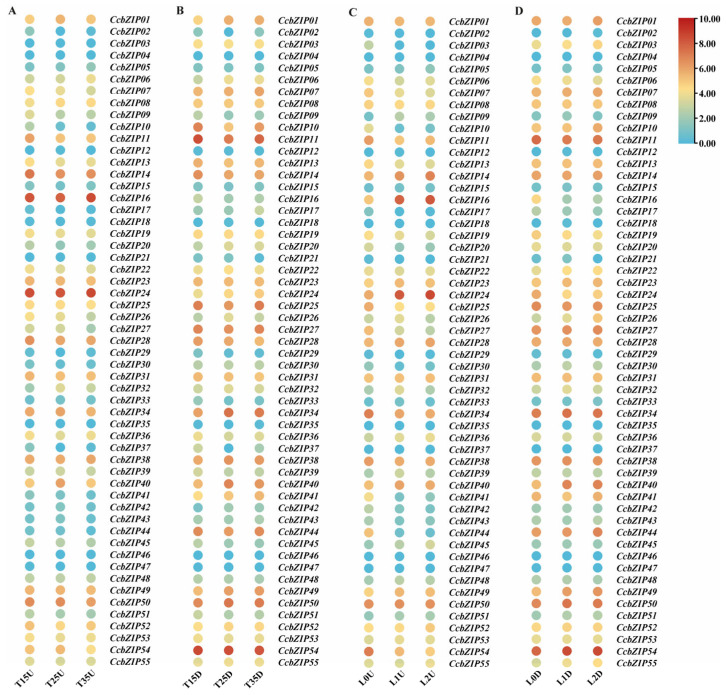
Expression profiles (log_2_ FPKM) of CcbZIP genes under abiotic stress. (**A**,**B**) Heatmaps showing expression of CcbZIP genes under different growth temperatures. T15: 15 °C; T25: 25 °C; T35: 35 °C; U: aerial tissues; D: subterranean tissues. (**C**,**D**) Heatmaps showing expression of CcbZIP genes under different light intensities.

To elucidate the transcriptional response of *CcbZIP* genes to environmental stimuli, we analyzed their expression profiles under varying temperature and light conditions using transcriptome data (Figure 6). FPKM-based heatmaps revealed that the expression of *CcbZIP07*, *CcbZIP08*, *CcbZIP26*, and *CcbZIP27* was notably influenced by temperature, whereas *CcbZIP07*, *CcbZIP09*, *CcbZIP13*, *CcbZIP14*, *CcbZIP16*, *CcbZIP20*, *CcbZIP24*, *CcbZIP34*, *CcbZIP45*, and *CcbZIP54* responded to different light intensities.

**Figure 7:**
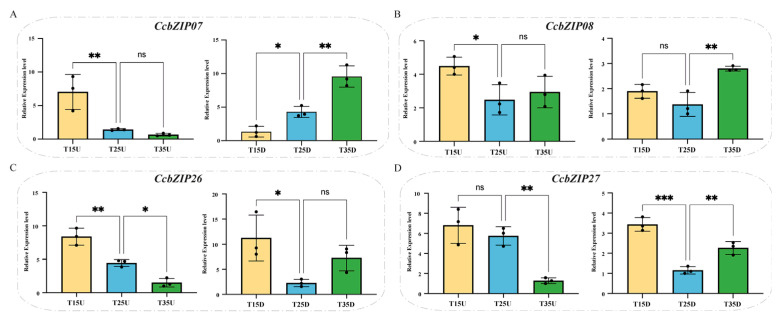
Expression patterns of selected CcbZIP genes under different temperature conditions. qRT-PCR analysis of (**A**) *CcbZIP07*, (**B**) *CcbZIP08*, (**C**) *CcbZIP26*, and (**D**) *CcbZIP27* in aerial (**left**) and subterranean (**right**) tissues. Data are presented as mean ± SD (*n* = 3). Asterisks indicate significant differences compared to the control (25 °C) at the same time point (* *p* < 0.05; ** *p* < 0.005; *** *p* < 0.0005).

We further validated these patterns using qRT-PCR under temperature stress (Figure 7). *CcbZIP07* expression was up-regulated in aerial tissues (U) at 15 °C and in subterranean tissues (D) at 35 °C. *CcbZIP08* showed no significant changes across temperature treatments. CcbZIP26 was up-regulated throughout the plant under low temperature (15 °C), suggesting a systemic cold response. *CcbZIP27* was down-regulated in U at 35 °C but up-regulated in D under both 15 °C and 35 °C, with a more pronounced induction under cold conditions.

**Figure 8:**
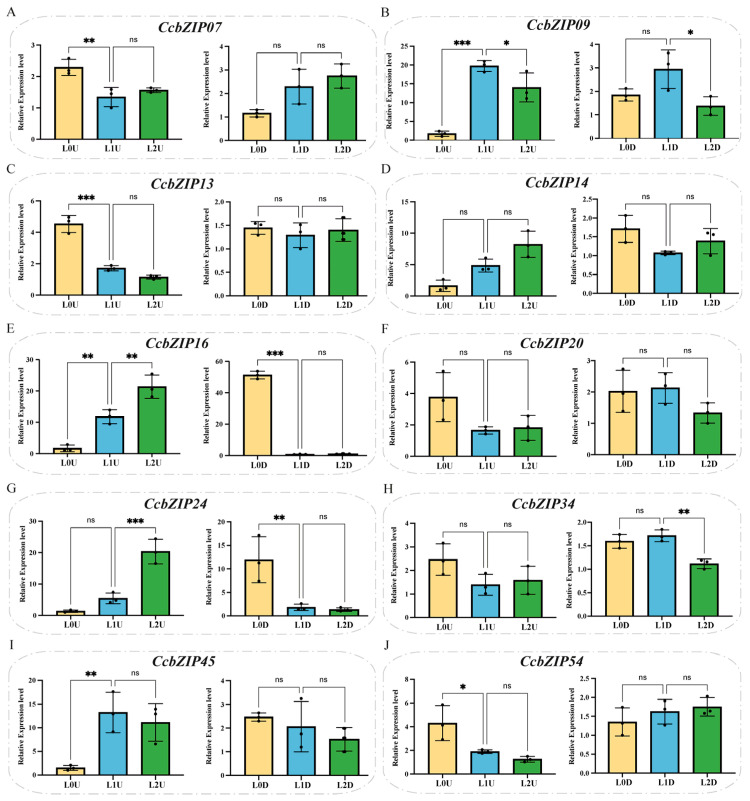
Expression patterns of selected CcbZIP genes under different light conditions. qRT-PCR analysis of (**A**) *CcbZIP07*, (**B**) *CcbZIP09*, (**C**) *CcbZIP13*, (**D**) *CcbZIP14*, (**E**) *CcbZIP16*, (**F**) *CcbZIP20*, (**G**) *CcbZIP24*, (**H**) *CcbZIP34*, (**I**) *CcbZIP45*, and (**J**) *CcbZIP54* in aerial (**left**) and subterranean (**right**) tissues under varying light intensities. Data are presented as mean ± SD (*n* = 3). Asterisks represent significant differences compared to the control (L1) at the same time point (* *p* < 0.05; ** *p* < 0.005; *** *p* < 0.0005).

Under light stress, tissue-specific expression patterns were also evident (Figure 8). *CcbZIP07* was induced in U under L0 intensity but unaffected in D. *CcbZIP09* was down-regulated in all tissues under L2. *CcbZIP13* was up-regulated in U under L0, while *CcbZIP14* remained unchanged. *CcbZIP16* decreased in U under L0 but increased under L2; in D, it increased under L0 and was stable under L2. *CcbZIP20* showed no significant response. *CcbZIP24* was up-regulated in U under L2 and in D under L0. *CcbZIP34* was suppressed in D under L2. *CcbZIP45* decreased in U under L0, and *CcbZIP54* increased in U under the same condition; neither gene showed significant changes in D. Collectively, these results demonstrate that *CcbZIP* genes exhibit distinct and tissue-specific expression patterns under temperature and light stresses, suggesting their potential roles in mediating environmental adaptation in *Coptis chinensis*.

## 3. Discussion

Our genome-wide analysis successfully identified and characterized 55 bZIP transcription factors (TFs) in the non-model medicinal plant *Coptis chinensis*. Phylogenetic classification revealed that the CcbZIP family is organized into 12 subfamilies, exhibiting notable divergence from the model plant *A. thaliana*. Two key observations underscore the unique evolutionary trajectory of this gene family in *C. chinensis*: the absence of the L and O subfamilies, and the presence of two orphan genes, *CcbZIP30* and *CcbZIP33*, which did not cluster with any established subfamily. The absence of entire subfamilies suggests significant lineage-specific adaptation of the bZIP functional landscape in *C. chinensis*. In Arabidopsis and other plants, the L and O subfamilies are often implicated in specific stress responses and developmental processes. Their loss in *C. chinensis* may indicate a rewiring of regulatory networks, potentially driven by selective pressures to optimize the production of specialized metabolites, such as the isoquinoline alkaloid berberine. Conversely, the marked expansion of subfamilies A and I—known to be involved in stress signaling and plant development—suggests that these functions are of critical importance in *C. chinensis*.

A particularly intriguing finding is the identification of *CcbZIP30* and *CcbZIP33*. As orphan genes, they may represent rapidly evolving, species-specific TFs that have acquired novel functions [34]. Given the rich and unique alkaloid profile of *C. chinensis*, it is tempting to speculate that these genes could be involved in the regulation of its specialized secondary metabolism, a compelling hypothesis that warrants further functional investigation.

Supporting their potential roles in environmental adaptation, our expression analysis revealed that numerous *CcbZIP* genes respond dynamically to temperature and light stresses in a tissue-specific manner. For instance, *CcbZIP26* was systemically up-regulated under cold stress (15 °C), while *CcbZIP27* exhibited contrasting regulation between aerial and subterranean tissues, suggesting complex, tissue-specific roles in temperature response. Similarly, several genes, including *CcbZIP07*, *CcbZIP16*, and *CcbZIP24*, showed distinct expression patterns under different light intensities. These tissue-specific expression profiles strongly imply that CcbZIP TFs are integral components of the signaling networks that mediate environmental perception and adaptive responses in *C. chinensis*.

In conclusion, this study provides the first systematic characterization of the bZIP family in *C. chinensis*. Our results not only elucidate the evolutionary dynamics of this important gene family in a medicinal plant but also pinpoint key candidate genes—particularly the expanded A and I subfamilies and the orphan genes *CcbZIP30* and *CcbZIP33*—for future research. Functional validation of these candidates will be crucial for understanding their specific roles in *C. chinensis* growth, stress adaptation, and, most importantly, the regulation of its valuable medicinal compounds.

## 4. Materials and Methods

### 4.1. Plant Materials and Stress Treatments

The *Coptis chinensis* cv. “Wei Lian” used in this study was employed. Two-year-old seedlings were collected from the Huangshui Town *C. chinensis* cultivation base in Shizhu County, Chongqing Municipality [35]. The seedlings were transplanted into soil in a growth chamber under controlled conditions: temperature of 26 °C (light)/20 °C (dark), relative humidity of approximately 60%, and a photoperiod of 16 h light/8 h darkness. After three weeks of acclimatization, when the seedlings had established and showed vigorous growth, uniformly growing seedlings were selected.

After acclimatization, uniformly growing seedlings were subjected to abiotic stress treatments for 48 h. For temperature treatments, plants were transferred to chambers set at constant temperatures of 15 °C (low temperature), 25 °C (control), or 35 °C (high temperature) during both light and dark periods, while maintaining the same light intensity and photoperiod as the acclimatization stage. For light stress treatments, plants were exposed to different light intensities (measured at the canopy level) using white LED lights: 476lx (low light, L0), 2060lx (normal light, L1), or 8340lx (high light, L2), while maintaining the temperature at 25 °C and the original photoperiod. Following treatment, aerial parts (U) and subterranean parts (D) were harvested separately, immediately frozen in liquid nitrogen, and stored at −80 °C for subsequent RNA extraction. For each treatment, three biological replicates were collected, each consisting of a pool of three individual plants.

### 4.2. Identification and Sequence Analysis of CcbZIP Genes

The protein sequences of 50 *Arabidopsis thaliana* bZIP transcription factors were used as queries to perform a BLASTP (version 2.17.0+) search (E-value ≤ 1 × 10^−5^) against the *C. chinensis* protein database (BioProject: SAMN15658057) using TBtools software (version 2.303). Simultaneously, the Hidden Markov Model (HMM) profiles for the bZIP domain (PF00170 and PF07716) were retrieved from the PFAM database and used to search the same protein database using HMMER 3.0 (E-value ≤ 1 × 10^−5^). All candidate sequences obtained from both searches were merged, and redundant sequences were removed. The presence of the conserved bZIP domain in each candidate was further verified using the NCBI Conserved Domain Database (CDD) and SMART database. The identified non-redundant genes were systematically renamed from *CcbZIP1* to *CcbZIP55* based on their ascending physical positions from chromosome 1 to 9. The physicochemical properties of the deduced CcbZIP proteins, including molecular weight and theoretical isoelectric point (pI), were predicted using the ExPASy ProtParam tool (https://web.expasy.org/protparam/, accessed on 17 December 2025). Subcellular localization was predicted using CELLO v2.5 [36].

### 4.3. Phylogenetic, Gene Structure, Conserved Motif Analysis, Chromosomal Distribution and Synteny Analysis

Multiple sequence alignment of the full-length amino acid sequences of the 55 identified CcbZIPs and 50 AtbZIPs was performed using ClustalW (version 2.1) with default parameters (Gap opening penalty = 10, Gap extension penalty = 0.2; and the alignment results were manually checked to correct obvious misalignments). A phylogenetic tree was constructed using the Maximum Likelihood (ML) method in MEGA7.0 software with the JTT substitution model and 1000 bootstrap replicates. The amino acid sequence of all genes was list in Table S1. The CcbZIP proteins were classified into subfamilies based on the established classification of their *A. thaliana* orthologs. The exon–intron structures of the CcbZIP genes were visualized based on the genome annotation file using the Gene Structure Display Server (GSDS). Conserved protein motifs were identified using the online MEME suite (version 5.5.8) with the following parameters: maximum number of motifs, 10; optimum motif width, 6 to 50 amino acids [37]. The identified motifs were annotated by searching against the NCBI-CDD database and visualized using TBtools [38]. The physical positions of the CcbZIP genes on the chromosomes were mapped using TBtools software. Gene duplication events were analyzed using the Multiple Collinearity Scan Toolkit (MCScanX, version 1.0) with default parameters [39].

### 4.4. RNA Extraction, Transcriptome Sequencing, and qRT-PCR Analysis

Total RNA was extracted from approximately 100 mg of ground tissue using TRIzol reagent (Invitrogen, Waltham, MA, USA) according to the manufacturer’s instructions. Genomic DNA was removed by treatment with DNase I (Takara, Kyoto, Japan). RNA integrity was verified using 1.5% agarose gel electrophoresis. For transcriptome sequencing, cDNA library construction and Illumina sequencing were performed by a commercial service (e.g., Novogene/BGI). Fastp (version 0.18.0) software was used to control the quality of raw RNA-seq data and remove the adapter [40]. The resulting clean reads were mapped to the *C. chinensis* reference genome, and gene expression levels were calculated as Fragments Per Kilobase of transcript per Million mapped reads (FPKM) using StringTie v1.3.1 [41,42].

For qRT-PCR validation, first-strand cDNA was synthesized from 1 μg of total RNA using the PrimeScript™ RT reagent kit with gDNA Eraser (Takara, Japan). Gene-specific primers were designed using Primer-BLAST (https://www.ncbi.nlm.nih.gov/tools/primer-blast/, accessed on 17 December 2025) to span exon-exon junctions, and primer specificity was verified by both 1.5% agarose gel electrophoresis (single clear band) and melt curve analysis (single peak) (Supplementary Table S2 for all primer sequences). The *C. chinensis* actin gene was used as an internal control [35]. qRT-PCR was performed in a 20 μL reaction volume containing 10 μL of 2× qPCR MasterMix (Applied Biological Materials, Richmond, BC, Canada), 0.8 μL of each primer (10 μM), 2 μL of 1:10 diluted cDNA, and 6.4 μL of nuclease-free water. The reactions were run on a CFX96 Touch Real-Time PCR Detection System (Bio-Rad, Hercules, CA, USA) with the following thermal cycling protocol: initial denaturation at 95 °C for 30 s, followed by 40 cycles of 95 °C for 5 s and 60 °C for 30 s. A melt curve analysis (65 °C to 95 °C with increments of 0.5 °C) was performed to confirm amplification specificity. All reactions were performed with three biological and three technical replicates. Relative gene expression levels were calculated using the 2^−ΔΔCt^ method [43]. Statistical significance was determined by one-way ANOVA using GraphPad Prism version 10.0 (* *p* < 0.05; ** *p* < 0.005; *** *p* < 0.0005) [44].

## 5. Conclusions

This study presents the first genome-wide identification and analysis of the bZIP transcription factor family in the medicinal plant *Coptis chinensis*. We identified 55 CcbZIP genes and classified them into 12 subfamilies based on phylogenetic relationships with *A. thaliana*. The absence of the L and O subfamilies, common in model plants, and the discovery of two putative orphan genes (*CcbZIP30* and *CcbZIP33*) highlight the unique evolutionary path of the bZIP family in this species. Furthermore, the distinct distribution of CcbZIP members, with significant expansions in stress-associated subfamilies A and I, suggests specific functional adaptations. Expression profiling confirmed that many CcbZIPs are responsive to temperature and light stresses in a tissue-specific manner. Collectively, these findings establish a crucial foundation for future functional studies aimed at elucidating the roles of CcbZIPs in the environmental adaptation and, potentially, the regulation of valuable benzylisoquinoline alkaloid biosynthesis in *C. chinensis*.

## Figures and Tables

**Table 1:** Characterisitic of CcbZIPS.

Gene Name	Gene ID	Chr	Chromosome Location	Gene Length (bp)	ORF Length (aa)	Deduced Protein				Subcellular Location
						Size (aa)	MW (kDa)	PI	GRAVY	
*CcbZIP01*	evm.model.Scaffold_63.37	6	68351006–68364660	13,654	242	81	9563.17	11.44	−0.932	Nuclear
*CcbZIP02*	evm.model.Scaffold_124.235	3	18006083–18007384	1301	848	283	31,878.16	6.37	−0.913	Nuclear
*CcbZIP03*	evm.model.Scaffold_123.165	6	93895910–93901866	5956	1252	420	47,214.42	6.69	−0.541	Nuclear
*CcbZIP04*	evm.model.Scaffold_65.11	3	22686753–22693929	4713	625	209	24,202.92	9.65	−0.506	Cytoplasmic
*CcbZIP05*	evm.model.Scaffold_56.68	6	1542536–1546055	3519	1004	335	37,011.98	5.84	−0.704	Nuclear
*CcbZIP06*	evm.model.Scaffold_128.187	8	88687098–88688981	1883	667	222	25,630.64	8.57	−0.919	Nuclear
*CcbZIP07*	evm.model.Scaffold_175.551	3	85971174–85979810	8636	941	315	35,617.36	6.64	−0.49	Nuclear
*CcbZIP08*	evm.model.Scaffold_161.2	2	3556701–3557183	2609	894	298	33,482.61	7.8	−0.881	Nuclear
*CcbZIP09*	evm.model.Scaffold_204.1	2	4085267–4090711	3335	1706	569	62,560.2	6.78	−0.895	Nuclear
*CcbZIP10*	evm.model.Scaffold_84.32	2	86525851–86526291	440	440	146	16,747.67	8.02	−0.784	Nuclear
*CcbZIP11*	evm.model.Scaffold_24.21	9	65115883–65116317	1659	473	157	17,965.1	6.29	−0.817	Nuclear
*CcbZIP12*	evm.model.Scaffold_165.276	2	12928906–12929914	1008	834	278	30,923.83	8.43	−0.915	Nuclear
*CcbZIP13*	evm.model.Scaffold_145.55	2	33619210–33623955	4745	1281	428	46,805.05	5.04	−0.711	Nuclear
*CcbZIP14*	evm.model.Scaffold_20.387	5	65762806–65763294	488	488	162	18,185.72	6.75	−0.466	Nuclear
*CcbZIP15*	evm.model.Scaffold_356.10	8	83877338–83884882	7544	2561	855	93,518.33	4.82	−0.619	Nuclear
*CcbZIP16*	evm.model.Scaffold_62.65	7	67370827–67376377	5550	470	157	18,044.1	9.52	−1.246	Nuclear
*CcbZIP17*	evm.model.Scaffold_27.71	7	85094226–85094804	578	578	192	22,328.99	6.06	−0.759	Nuclear
*CcbZIP18*	evm.model.Scaffold_106.121	2	10277150–10278158	1008	834	278	30,923.83	8.43	−0.915	Nuclear
*CcbZIP19*	evm.model.Scaffold_106.478	2	251653–259758	8105	1372	460	50,789.87	8.75	−0.603	Nuclear
*CcbZIP20*	evm.model.Scaffold_191.13	--	97202–101121	3919	1214	405	44,374.16	5.51	−0.613	Nuclear
*CcbZIP21*	evm.model.Scaffold_252.40	3	13184165–13189401	5236	1098	368	42,110.74	6.14	−0.498	Nuclear
*CcbZIP22*	evm.model.Scaffold_161.83	2	2983894–2986739	2845	2059	686	74,454.55	7.18	−0.524	Endoplasmic Reticulum
*CcbZIP23*	evm.model.Scaffold_360.34	4	16145365–16151420	6055	1230	413	43,786.68	8.31	−0.792	Nuclear
*CcbZIP24*	evm.model.Scaffold_46.103	1	70302836–70303670	834	542	181	20,387.9	6.99	−0.809	Nuclear
*CcbZIP25*	evm.model.Scaffold_194.41	1	34592896–34597504	4608	1281	428	47,129.15	5.64	−0.636	Nuclear
*CcbZIP26*	evm.model.Scaffold_38.133	9	50051894–50055400	3506	1161	388	44,472.64	9.13	−0.609	Nuclear
*CcbZIP27*	evm.model.Scaffold_203.105	7	21177099–21183198	6099	1494	501	55,899.55	6.25	−0.588	Nuclear
*CcbZIP28*	evm.model.Scaffold_41.681	4	90980279–90983595	3316	864	288	31,318.5	4.78	−0.837	Nuclear
*CcbZIP29*	evm.model.Scaffold_370.16	--	123764–128992	5228	1323	443	50,180.74	5.22	−0.403	Nuclear
*CcbZIP30*	evm.model.Scaffold_4.341	3	103306282–103317534	11,252	2178	731	81,352.76	5.28	−0.526	Nuclear
*CcbZIP31*	evm.model.Scaffold_78.543	8	93164141–93170657	6516	1209	406	43,075.3	5.6	−0.893	Nuclear
*CcbZIP32*	evm.model.Scaffold_226.12	2	2736440–2739282	2842	2056	685	74,336.37	7.18	−0.52	Cytoplasmic
*CcbZIP33*	evm.model.Scaffold_226.30	2	2628293–2633196	4903	914	307	33,109.99	4.89	−0.457	Chloroplast
*CcbZIP34*	evm.model.Scaffold_41.632	4	90521302–90524839	3537	1076	359	39,393.77	6.34	−0.772	Nuclear
*CcbZIP35*	evm.model.Scaffold_187.200	3	66584201–66591229	7028	732	244	27,969.38	5.31	−0.802	Nuclear
*CcbZIP36*	evm.model.Scaffold_282.29	4	85802034–85805554	3520	1127	376	42,433.66	6.78	−1.045	Nuclear
*CcbZIP37*	evm.model.Scaffold_89.303	9	57192590–57193532	942	702	231	25,845.31	5.23	−0.756	Nuclear
*CcbZIP38*	evm.model.Scaffold_81.149	6	96334268–96345074	10,806	1324	444	48,989.11	5.91	−0.44	Nuclear
*CcbZIP39*	evm.model.Scaffold_371.5	--	73269–76031	2762	1106	369	41,307.57	6.5	−0.943	Nuclear
*CcbZIP40*	evm.model.Scaffold_72.208	2	9921150–9922803	1653	804	268	30,140.36	5.76	−0.432	Nuclear
*CcbZIP41*	evm.model.Scaffold_72.137	2	9254146–9254628	482	482	160	18,879.41	11.04	−0.831	Nuclear
*CcbZIP42*	evm.model.Scaffold_34.97	3	3647551–3651470	3919	1214	405	44,422.25	5.59	−0.607	Nuclear
*CcbZIP43*	evm.model.Scaffold_67.62	6	16899934–16900026	2600	1307	436	47,732.88	8.54	−0.918	Nuclear
*CcbZIP44*	evm.model.Scaffold_67.72	6	16703040–16703186	10,243	1431	480	54,036.08	8.88	−0.579	Nuclear
*CcbZIP45*	evm.model.Scaffold_31.662	2	4956546–4959921	3375	1706	569	62,557.24	6.78	−0.888	Nuclear
*CcbZIP46*	evm.model.Scaffold_11.13	8	67776316–67781705	1657	315	105	11,959.71	10.28	−0.69	Chloroplast
*CcbZIP47*	evm.model.Scaffold_110.91	1	75592581–75595718	3137	879	294	33,138.64	6.83	0.023	Plasma Membrane
*CcbZIP48*	evm.model.Scaffold_110.382	2	17308158–17319069	10,911	2674	893	97,509.61	4.79	−0.63	Nuclear
*CcbZIP49*	evm.model.Scaffold_1.8	--	55368–59792	7079	1183	397	41,833.24	6.11	−0.696	Nuclear
*CcbZIP50*	evm.model.Scaffold_109.640	4	71801476–71806600	5124	1465	489	53,474.72	9.41	−0.791	Nuclear
*CcbZIP51*	evm.model.Scaffold_273.40	7	11663351–11669409	6058	1064	355	38,922.79	5.52	−0.794	Nuclear
*CcbZIP52*	evm.model.Scaffold_232.8	6	17932846–17933243	5953	1542	515	57,476.6	8.13	−0.797	Nuclear
*CcbZIP53*	evm.model.Scaffold_93.79	4	56509773–56510094	4200	1847	616	68,403.26	7.77	−0.997	Nuclear
*CcbZIP54*	evm.model.Scaffold_15.169	2	59307068–59307502	434	434	144	16,569.83	5.22	−0.576	Nuclear
*CcbZIP55*	evm.model.Scaffold_264.349	7	397066–405341	8275	1372	460	50,764.88	8.43	−0.59	Nuclear

## Data Availability

The data presented in this study are available on request from the corresponding author.

## Supplementary Materials

The following supporting information can be downloaded at: https://www.mdpi.com/article/10.3390/ijms27010431/s1.

## Abbreviations

The following abbreviations are used in this manuscript:

bZIP	basic leucine zipper
qRT-PCR	Quantitative Real-time PCR
ABA	abscisic acid
HMM	Hidden Markov Model
CDD	Conserved Domain Database
